# Efficacy of Atezolizumab, Bevacizumab, Carboplatin, and Paclitaxel Therapy in Patients With Genetic Alterations in Non‐Small Cell Lung Cancer

**DOI:** 10.1111/1759-7714.70162

**Published:** 2025-09-17

**Authors:** Tsunehiro Tanaka, Motohiro Tamiya, Akito Miyazaki, Kiyohide Komura, Shun Futamura, Takahisa Kawamura, Kei Kunimasa, Takako Inoue, Kazumi Nishino

**Affiliations:** ^1^ Department of Thoracic Oncology Osaka International Cancer Institute Osaka Osaka Japan

**Keywords:** ABCP therapy, *ALK* fusion, bevacizumab, *EGFR* mutation, *KRAS* mutation

## Abstract

**Background:**

The IMpower150 trial demonstrated the efficacy of atezolizumab, bevacizumab, carboplatin, and paclitaxel (ABCP) therapy in epidermal growth factor receptor ( *EGFR* )‐mutated non‐small cell lung cancer (NSCLC). However, its efficacy in patients with NSCLC harboring other genetic alterations in real‐world settings remains unclear. This study aimed to retrospectively evaluate the efficacy of ABCP therapy in patients with NSCLC harboring other genetic alterations.

**Methods:**

We retrospectively analyzed 61 patients with advanced NSCLC (33 with *EGFR* mutations: EGFR group and 28 with other genetic alterations: other group) who received ABCP therapy between January 2019 and December 2023 at a single institution in Japan, and evaluated efficacy and toxicities.

**Results:**

Most baseline characteristics were similar except treatment timing (*p* < 0.001) in both groups. The median progression‐free survival (PFS) was 4.5 vs. 5.1 months (*p* = 0.663), and the objective response rate (ORR) was 45.5% vs. 50.0% (*p* = 0.77) between the EGFR and other groups. In multivariate analysis, PD‐L1 expression ≥ 50% was independently associated with longer PFS (HR 0.23, *p* < 0.001). Grade ≥ 3 adverse events were manageable and occurred at similar rates (51.5% vs. 53.6%) between the EGFR and other groups, and discontinuation was low (9.8%). Subgroup analysis for patients with KRAS mutation (*n* = 14) and anaplastic lymphoma kinase (ALK) fusion (*n* = 6) showed trends consistent with the overall cohort.

**Conclusions:**

ABCP therapy demonstrated efficacy and manageable toxicity in NSCLC patients in both groups. Notably, those with high PD‐L1 expression (≥ 50%) may derive greater PFS benefit. Further confirmation in larger prospective trials is warranted.

## Graphical abstract

This retrospective study evaluated 61 patients with advanced NSCLC harboring genetic alterations treated with ABCP therapy. Efficacy and safety were comparable between the EGFR (*n* = 33) and other (*n* = 28) groups. Notably, patients with high PD‐L1 expression (≥ 50%) derived a significant progression‐free survival benefit.

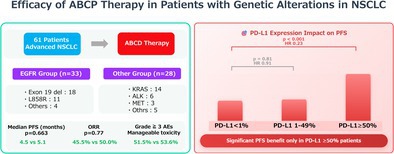

## 1 Introduction

Lung cancer remains a leading cause of cancer‐related death worldwide, with an estimated 1.8 million deaths in 2022 [1]. In non‐small cell lung cancer (NSCLC), which accounts for most lung cancer cases, genetic alterations play a critical role in both pathogenesis and response to treatment. The identification of targetable genetic alterations is essential for the treatment of advanced‐stage NSCLC and for determining appropriate systemic chemotherapy. Approximately 50% to 60% of patients with lung adenocarcinoma harbor potentially targetable genetic alterations [2, 3]. While molecularly targeted therapies have shown remarkable efficacy in patients with specific genetic alterations, resistance inevitably develops, necessitating alternative treatment strategies [4, 5]. Furthermore, therapeutic options are limited in patients after exhaustion of targeted therapies.

Recently, immunotherapy targeting the programmed death‐1 checkpoint pathway has provided a therapeutic option for patients with advanced NSCLC. Immunotherapy, whether as monotherapy or in combination with chemotherapy, has improved clinical outcomes compared with standard chemotherapy regimens [6]. However, patients with genetic alterations such as epidermal growth factor receptor (*EGFR*) mutations and anaplastic lymphoma kinase (*ALK*) fusions have shown limited response to immune checkpoint inhibitor (ICI) monotherapy [7, 8, 9]. Additionally, the KEYNOTE‐789 trial suggested that combination immunotherapy had limited efficacy in patients with *EGFR*‐positive NSCLC [10].

In contrast, the IMpower150 trial, which evaluated the efficacy and safety of atezolizumab, bevacizumab, carboplatin, and paclitaxel (ABCP) therapy, demonstrated improved overall survival (OS) and progression‐free survival (PFS) compared to the standard of care. In the trial, subset analysis suggested efficacy even in patients with *EGFR* mutations [11]. Furthermore, two phase III clinical trials (the ORIENT trial and ATTLAS) have shown that the addition of an anti‐angiogenic agent to combination immunotherapy can enhance efficacy, even in patients with *EGFR* mutations [12, 13]. However, the efficacy of this four‐drug combination (ABCP) therapy in patients with advanced NSCLC with other genetic alterations remains unclear.

Therefore, we retrospectively evaluated patients treated with ABCP therapy at our institution to evaluate efficacy and safety and to explore whether outcomes differed between patients with EGFR mutations: EGFR group and those with other genetic alterations (ALK, KRAS, MET, and others): Other group.

## 2 Methods

### 2.1 Study Design

We conducted a single‐center, retrospective cohort study in Japan. Clinical data of the patients were retrospectively extracted from their medical records and added to a database. Sample size calculation based on hypothesis testing was not performed because this was a retrospective observational study.

This study was approved by the Ethics Committee of the Osaka International Cancer Institute (Approval No. 25030‐2). Informed consent was not required due to the retrospective nature of the study, and an opt‐out method was used, allowing patients and their families to refuse to participate in the study.

### 2.2 Patient Selection

This study included patients with advanced or postoperative recurrent NSCLC who received ABCP therapy, either after tyrosine kinase inhibitor (TKI) treatment or as first‐line treatment, at the Osaka International Cancer Institute between January 2019 and December 2023. Clinical characteristics at the initiation of ABCP therapy were obtained, including sex, age, smoking history, programmed death ligand 1 (PD‐L1) expression, histologic type, genetic alteration status, and Eastern Cooperative Oncology Group (ECOG) performance status (PS) through a retrospective review of medical records. Of the 62 patients who received ABCP‐based therapy during the study period, one patient was excluded due to omission of bevacizumab as determined by the treating physician. The final analysis included 61 patients. A flow diagram of patient selection is provided in Figure S1. Genetic alterations, including *EGFR*, *ALK*, *KRAS*, *MET*, and others, were evaluated using clinically approved genetic testing methods available in routine practice in Japan. These included PCR‐based assays (e.g., cobas *EGFR* Mutation Test v2), immunohistochemistry (e.g., Ventana *ALK* [D5F3] IHC with confirmatory testing as needed), and multigene next‐generation sequencing panels (e.g., Oncomine Dx Target Test) covered by the national health insurance system.

### 2.3 Assessment of Efficacy and Toxicity

Progression‐free survival (PFS) was determined by investigator assessment according to the Response Evaluation Criteria in Solid Tumors v1.1 (RECIST v1.1) [14]. PFS was defined as the time from the first dose of ABCP to documented disease progression or death, whichever occurred first. Censoring was applied at the last tumor assessment or clinical follow‐up for those without progression, at the date of discontinuation if ABCP was stopped for reasons other than progression or death, and at the last tumor assessment prior to the initiation of subsequent systemic therapy. OS was defined as the time from the first dose of ABCP to death from any cause. Patients alive at the data cutoff were censored at the last known date alive; subsequent therapies were ignored. Objective response rate (ORR) and disease control rate (DCR) were assessed per RECIST v1.1. In the primary analysis, patients with non‐evaluable response (NE) were excluded from the denominator. As a sensitivity analysis, we additionally calculated ORR and DCR including NE as non‐responders.

Adverse events were graded per CTCAE v5.0. irAEs were specifically captured for at least 720 days after ABCP initiation. Survival outcomes were followed until December 31, 2024.

### 2.4 Statistical Analysis

Categorical variables were analyzed using Fisher's exact test, and continuous variables were analyzed using the Wilcoxon rank‐sum test. PFS and OS were estimated using the Kaplan–Meier method, and differences between patient groups were compared using the log‐rank test. To identify factors associated with PFS, univariate and multivariate Cox proportional hazards models were performed. Variables with a *p*‐value < 0.1 in univariate analysis were included in the multivariate model. The following baseline covariates were evaluated: age (≥ 70 vs. < 70 years), sex, ECOG performance status (0–1 vs. ≥ 2), smoking history (ever vs. never), PD‐L1 expression (< 1%, 1%–49%, ≥ 50%), treatment timing (post‐TKI vs. first‐line), and genetic alteration group (EGFR group vs. other group). Patients with unknown PD‐L1 status were excluded from analyses involving PD‐L1 expression, including Cox regression. Hazard ratios (HRs) with corresponding 95% confidence intervals (CIs) and *p*‐values were calculated. All statistical analyses were performed using EZR (Saitama Medical Center, Jichi Medical University, Saitama, Japan), and a *p*‐value < 0.05 was considered statistically significant. All subgroup comparisons, including pairwise log‐rank tests (e.g., EGFR vs. KRAS or ALK), were exploratory and not pre‐specified in the study protocol.

## 3 Results

### 3.1 Patients' Characteristics

Overall, 61 patients were enrolled in this study between January 2019 and December 2023. Enrolled patients were categorized into the EGFR group (including 33 patients with *EGFR* mutations) and other group (including 28 patients with other genetic alterations: The detailed distribution of genetic subtypes is summarized in Table S1).

The characteristics of the patients are shown in Table 1. Between the EGFR group and the other group, there were similar distributions: the median age (63 years [range: 41–78] vs. 64 years [range: 42–75], *p* = 0.643), sex (female: 63.6% vs. 53.6%, *p* = 0.447), smoking history (ever smoker: 57.6% vs. 57.1%, *p* = 1.000), and ECOG PS (0–1: 84.8% vs. 85.7%, *p* = 1.000). All patients had adenocarcinoma histology. However, treatment timing differed significantly between groups (*p* < 0.001). In the EGFR group, 93.3% (31/33) of patients received ABCP after TKI failure. Two patients did not receive EGFR‐TKIs before ABCP: one had an *EGFR* exon 20 insertion, which is generally associated with resistance to currently approved EGFR‐TKIs; the other was initially diagnosed as *EGFR* mutation‐negative at a previous institution, but a re‐biopsy performed after transfer to our hospital confirmed the mutation after ABCP had already been initiated. In contrast, 71.4% (20/28) of patients in the other group received ABCP as first‐line treatment, while the remaining 28.6% (8/28) received it after progression on prior targeted therapies (*p* < 0.001). These included ALK inhibitors (alectinib, brigatinib), MET inhibitors (tepotinib; in one case, glumetinib administered within a clinical trial), a RET inhibitor (selpercatinib), and BRAF/MEK inhibitors (dabrafenib plus trametinib). Among the patients with ALK rearrangements, two did not receive ALK‐TKI therapy prior to ABCP. In one case, ALK positivity was identified through re‐biopsy at our institution after ABCP initiation. In the other case, chemotherapy was started before the genetic testing results were available due to rapidly progressing disease. PD‐L1 status was evenly distributed between the groups (*p* = 0.619), even though seven patients in the EGFR group had unknown PD‐L1 expression status.

**TABLE 1:** Patient characteristics according to genetic alterations status.

Characteristics	EGFR group (*N* = 33)	Other group (*N* = 28)	*p*
Age, years			
Median (range)	63 (41–78)	64 (42–75)	0.643
Sex			0.447
Male	12 (36.4)	13 (46.4)	
Female	21 (63.6)	15 (53.6)	
ECOG PS			1
0–1	28 (84.8)	24 (85.7)	
≥ 2	5 (15.2)	4 (14.3)	
Smoking history			1
Never	14 (42.4)	12 (42.9)	
Ever	19 (57.6)	16 (57.1)	
Histology			
Adenocarcinoma	33 (100.0)	28 (100.0)	
PD‐L1 expression			0.619
< 1%	7 (26.9)	9 (32.1)	
1%–49%	9 (34.6)	12 (42.9)	
≥ 50%	10 (38.5)	7 (25.0)	
Unknown	7	0	
Prior TKI therapy			< 0.001
Yes	31 (93.9)	8 (28.6)	
No	2 (6.1)	20 (71.4)	
Genetic alterations			
EGFR mutation	33 (100.0)	—	
KRAS mutation	—	14 (50.0)	
ALK fusion	—	6 (21.4)	
MET alteration	—	3 (10.7)	
Others^a^	—	5 (17.9)	

Abbreviations: ECOG PS, Eastern Cooperative Oncology Group performance status; PD‐L1, programmed death ligand 1; TKI, tyrosine kinase inhibitor.

^a^ Others include *HER2* mutation (*n* = 1), *BRAF V600E* mutation (*n* = 1), *NRG1* fusion (*n* = 1), *RET* fusion (*n* = 1), and *ROS1* fusion (*n* = 1). Data are presented as *n* (%) unless otherwise specified.

### 3.2 Efficacy of ABCP

**FIGURE 1:**
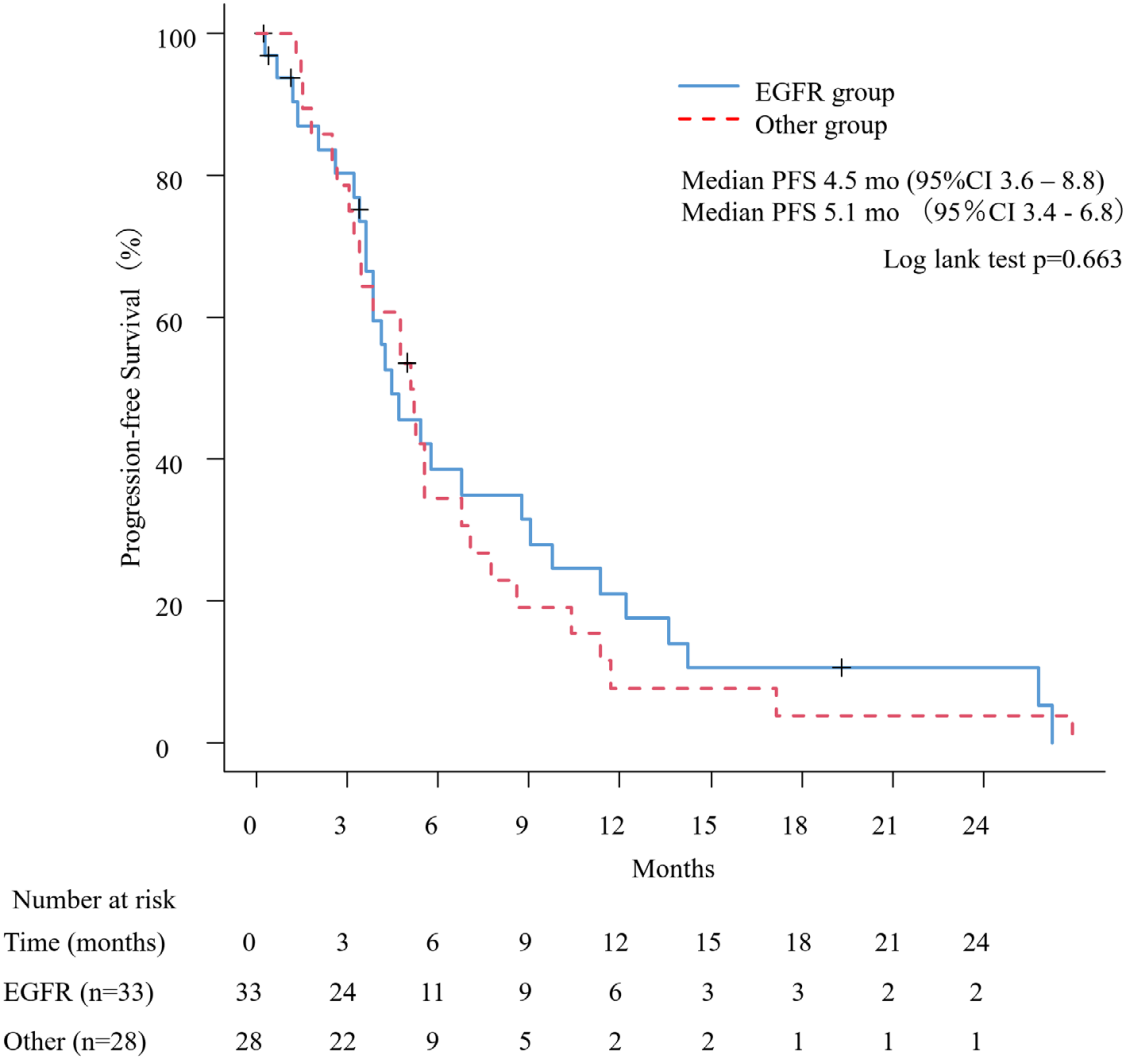
Kaplan–Meier curve for progression‐free survival (PFS) in patients receiving atezolizumab, bevacizumab, carboplatin, and paclitaxel (ABCP) therapy. The solid line represents patients with EGFR mutations (*n* = 33), while the dashed line represents patients with other genetic alterations (*n* = 28).

The median follow‐up duration, calculated using the reverse Kaplan–Meier method, was 30.5 months (95% confidence interval: 22.6–34.7). The median PFS was 4.5 months (95% confidence interval [CI]: 3.6–8.8) in the EGFR group compared to 5.1 months (95% CI: 3.4–6.8) in the other group (Log‐rank *p* = 0.663; Figure 1). The median OS was 18.3 months (95% CI: 11.5 − NA) in the EGFR group and 15.1 months (95% CI: 11.6–38.1) in the other group (log‐rank *p* = 0.978). Further subgroup analysis of non‐*EGFR* alterations focused on *KRAS* mutations and *ALK* fusions. Patients with KRAS mutations (*n* = 14) showed a median PFS of 5.4 months (95% CI: 3.4–8.6) and a median OS of 14.7 months (95% CI: 9.4–38.1). In the ALK fusion group (*n* = 6), the median PFS and OS were 4.1 months (95% CI: 1.5 − NA) and 24.9 months (95% CI: 4.2 − NA), respectively. No statistically significant differences were observed between these subgroups and the EGFR group (KRAS vs. EGFR: PFS *p* = 0.845, OS *p* = 0.917; ALK vs. EGFR: PFS *p* = 0.309, OS *p* = 0.994). Within the EGFR group, 18 patients had exon 19 deletion and 11 had L858R mutation. The median PFS was 4.2 months (95% CI: 3.6–8.7) for the exon 19 deletion group and 5.5 months (95% CI: 1.2–11.3) for the L858R group (*p* = 0.474). Kaplan–Meier curves for PFS by *EGFR* subtype are presented in Figure S2. Minor mutation cases (*n* = 4) were not analyzed due to limited sample size.

The ORR was 45.5% (15/33) in the EGFR group and 50.0% (14/28) in the other group (*p* = 0.77). The DCR was 72.7% (24/33) and 78.6% (22/28), respectively (*p* = 0.91). In a sensitivity analysis counting NE as non‐responders, ORR was 45.5% (15/33) vs. 50.0% (14/28) (*p* = 0.80) and DCR was 72.7% (24/33) vs. 78.6% (22/28) (*p* = 0.77), yielding no material change in conclusions.

### 3.3 Univariate and Multivariate Analyses of PFS

Univariate Cox proportional hazards analysis was performed to evaluate factors associated with PFS. Among the variables tested, PD‐L1 expression ≥ 50% was significantly associated with longer PFS (HR 0.247; 95% CI: 0.108–0.565; *p* < 0.001). In contrast, treatment timing (post‐TKI vs. first‐line; HR 1.325; 95% CI: 0.752–2.332; *p* = 0.33), genetic alteration group (EGFR group vs. Other group; HR 0.887; 95% CI: 0.518–1.522; *p* = 0.665), and other baseline factors were not significantly associated with PFS (Table 2).

**TABLE 2:** Univariate and multivariate Cox regression analyses for progression‐free survival (PFS).

Variable	Univariate analysis			Multivariate analysis		
	HR (95% CI)	*p*	*N*	HR (95% CI)	*p*	*N*
Age			61			54
≥ 70 years vs. < 70 years	0.887 (0.473–1.663)	0.708		0.751 (0.365–1.546)	0.437	
Sex			61			54
Male vs. female	0.897 (0.516–1.560)	0.701		1.192 (0.627–2.264)	0.592	
ECOG performance status			61			54
0–1 vs. ≥ 2	0.807 (0.358–1.822)	0.606		1.264 (0.451–3.545)	0.656	
Smoking history			61			
Ever vs. never	0.836 (0.479–1.456)	0.526		—	—	
PD‐L1 expression			54			54
≥ 50% vs. < 1%	0.247 (0.108–0.565)	< 0.001		0.228 (0.097–0.537)	< 0.001	
1%–49% vs. < 1%	0.999 (0.500–1.996)	0.998		0.912 (0.438–1.899)	0.805	
Treatment timing			61			
Post‐TKI vs. first‐line	1.325 (0.752–2.332)	0.33		—	—	
Genetic alteration			61			54
EGFR group vs. other group	0.887 (0.518–1.522)	0.665		0.868 (0.467–1.613)	0.654	

*Note:* Variables with *p* < 0.1 in univariate analysis were included in the multivariate model. Treatment line (first‐line vs. post‐TKI) was not significant in univariate analysis and was therefore not included in the multivariate model. *N* values differ due to exclusion of patients with unknown PD‐L1 status.

In multivariate analysis including age, sex, ECOG performance status, PD‐L1 expression, and genetic alteration group, only PD‐L1 expression ≥ 50% remained independently associated with improved PFS (HR 0.228; 95% CI: 0.097–0.537; *p* < 0.001). All other covariates were not statistically significant (Table 2).

### 3.4 Adverse Events Following ABCP Therapy

The safety profile was evaluated in all 61 patients (Table 3). Treatment‐related adverse events (TRAEs) of any grade were observed in all patients in both groups. Grade ≥ 3 TRAEs occurred in 17 (51.5%) patients in the EGFR group and 15 (53.6%) patients in the other group.

**TABLE 3:** Treatment‐related adverse events.

Adverse events	EGFR group (*n* = 33)		Other group (*n* = 28)	
	Any grade	Grade ≥ 3	Any grade	Grade ≥ 3
Any adverse events	33 (100.0)	17 (51.5)	28 (100.0)	15 (53.6)
Hematologic toxicities				
Neutropenia	21 (63.6)	9 (27.3)	14 (50.0)	8 (28.6)
Anemia	10 (30.3)	1 (3.0)	14 (50.0)	2 (7.1)
Thrombocytopenia	11 (33.3)	3 (9.1)	15 (53.6)	3 (10.7)
Non‐hematologic toxicities				
Fatigue	3 (9.1)	1 (3.0)	4 (14.3)	0 (0.0)
Peripheral neuropathy	8 (24.2)	2 (6.1)	12 (42.9)	0 (0.0)
Proteinuria	15 (45.5)	1 (3.0)	10 (35.7)	2 (7.1)
Hypertension	8 (24.2)	1 (3.0)	6 (21.4)	2 (7.1)
Immune‐related AEs (irAEs)				
Any irAEs	13 (39.4)	6 (18.2)	13 (46.4)	5 (17.9)
Treatment discontinuation due to AEs	4 (12.1)	—	2 (7.1)	—

*Note:* Data are presented as *n* (%). Adverse events were graded according to the National Cancer Institute Common Terminology Criteria for Adverse Events, version 5.0.

Abbreviation: EGFR, epidermal growth factor receptor.

Hematologic toxicities were the most common adverse events, with grade ≥ 3 events reported in 15 (45.5%) patients in the EGFR group and 11 (39.3%) patients in the other group. Grade ≥ 3 non‐hematologic toxicities were observed in seven (21.2%) patients in the EGFR group and eight (28.6%) patients in the Other group, respectively.

Immune‐related adverse events (irAEs) were observed in 13 (39.4%) and 13 (46.4%) patients in EGFR and Other groups, respectively, with grade ≥ 3 irAEs in 5 (15.2%) and 5 (17.9%). Rash, hypothyroidism, and pneumonitis were the most common events. Detailed profiles are provided in Table S3.

Treatment discontinuation due to AEs was required in four (12.1%) patients in the EGFR group and two (7.1%) patients in the other group. No treatment‐related deaths were observed.

## 4 Discussion

In this retrospective analysis, ABCP therapy showed activity in patients with EGFR mutations and in those with other genetic alterations; no statistically significant PFS difference was observed between groups. To our knowledge, this is the first report comparing outcomes among different genetic alterations in patients receiving ABCP therapy. Because OS is strongly influenced by mutation‐specific prognostic differences, our OS findings are reported descriptively, with treatment activity mainly inferred from PFS and ORR.

Patients with genetic alterations have generally been considered to have a limited response to immunotherapy. Furthermore, in patients with an EGFR mutation, some anti‐PD‐L1 antibody‐combined chemotherapy was found to be less beneficial. However, among the immune‐chemotherapy options, ABCP showed relatively favorable outcomes in *EGFR* mutation‐positive lung cancer. In our study, although no statistically significant difference in PFS was observed between the EGFR and Other groups, this does not imply equivalent efficacy. Given the limited sample size and differences in treatment settings, our findings should be interpreted with caution and viewed as hypothesis‐generating.

Based on the available evidence and our observations, we believed that there may be two potential explanations for these findings. One possible explanation involves the tumor microenvironment (TME) in NSCLC with certain genetic alterations, such as *EGFR* or *ALK*, which have been associated with reduced immunogenicity and often exhibit lower tumor mutational burden (TMB) and decreased neoantigen load [15, 16]. These immunological features are typically linked to diminished responsiveness to ICIs. However, the immune profiles of genetically altered tumors can be heterogeneous; for instance, *KRAS*‐mutant tumors often demonstrate relatively higher TMB, suggesting potential differences in ICI sensitivity across mutation subtypes [17].

Anti‐angiogenic therapy such as bevacizumab may serve as a strategy to modulate the TME. Normalizing aberrant vasculature can improve oxygenation and facilitate the trafficking of immune effector cells [18, 19]. Furthermore, anti‐angiogenic agents reportedly reduce immunosuppressive cell populations, such as regulatory T cells and myeloid‐derived suppressor cells, while enhancing the recruitment and activation of cytotoxic T lymphocytes [18, 20, 21]. Furthermore, the addition of immune checkpoint inhibitors, such as anti–PD‐L1 antibodies, further enhances the anti‐tumor immune response by blocking the interaction between PD‐L1 on tumor cells and PD‐1 on T cells. This blockade restores the activity of exhausted cytotoxic T lymphocytes, allowing them to effectively attack tumor cells. When combined with anti‐angiogenic agents like bevacizumab, which normalize tumor vasculature and modulate the immune microenvironment, checkpoint inhibitors may achieve greater efficacy due to improved T cell infiltration and activation within the tumor. These changes in the TME may contribute to overcoming, at least partially, the immune resistance observed in some genetically altered tumors, providing a plausible explanation for the observed efficacy of ABCP therapy even among genetically diverse subgroups.

Second, bevacizumab may play a role in overcoming resistance to molecular targeted therapies. Known resistance mechanisms include (1) secondary specific genetic alterations such as T790M that impede drug binding; (2) activation of alternative pathways, such as *MET* overexpression or amplification, vascular endothelial growth factor receptor 2 (*VEGFR2*) overexpression, HER2/HER3 signaling activation, and alterations in the PI3K/AKT signaling pathway; (3) histological changes such as transformation to small‐cell lung cancer and epithelial‐mesenchymal transition, which have been reported [22]. EGFR and vascular endothelial growth factor (VEGF) share common downstream signaling pathways (PI3K/AKT and RAS/RAF/ERK) and can function independently during oncogenesis and acquired therapeutic resistance, particularly in *EGFR*‐mutant NSCLC. Activated EGFR signaling increases VEGF through hypoxia‐independent mechanisms. Finally, during acquired resistance to EGFR TKIs, VEGF levels substantially increase even when EGFR phosphorylation remains suppressed. VEGF can promote tumor growth by activating PI3K/AKT and MAPK pathways independently of EGFR [23]. Furthermore, based on the research by Watanabe et al., *VEGFR2* expression is elevated across multiple oncogene‐driven NSCLCs, not just those with EGFR mutations [24]. VEGF pathway upregulation is a common feature across different oncogene‐driven lung cancers and may represent a shared resistance mechanism to targeted therapies. Inhibition of VEGF (bevacizumab) may be a promising therapeutic strategy not only for *EGFR*‐mutated NSCLC but also for other gene‐mutated NSCLC. In contrast, among the activation of alternative pathways, *MET* overexpression or amplification is one of the most well‐known mechanisms [23, 25, 26]. In bypass pathway activation, *MET* amplification leads to phosphorylation of ErbB3 (HER3), which activates PI3K/AKT and MEK/MAPK pathways, providing bypass signaling in the presence of EGFR‐TKIs. Beyond *EGFR* mutations, *MET* amplification also mediates resistance to TKIs targeting *ALK*, *ROS1*, and *RET* fusions, and the *KRAS* G12C mutation in lung cancer [27] Bevacizumab may help overcome *MET*‐mediated resistance by targeting the hepatocyte growth factor‐driven VEGF production and subsequent angiogenesis that supports tumor growth and therapeutic resistance [28].

The IMpower150 trial reported improved PFS with the addition of bevacizumab in patients with EGFR mutations; however, the final overall survival analysis did not show a statistically significant benefit in this subgroup, as the hazard ratio crossed 1.0 [11]. Similarly, in the ATTLAS trial, while PFS was improved with ABCP compared to chemotherapy, no OS advantage was observed [13]. In our study, ABCP therapy demonstrated modest efficacy in EGFR‐mutated patients, with a median PFS of 4.5 months and OS of 18.3 months. These outcomes are numerically lower than those in the IMpower150 and ATTLAS trials, and given the absence of a chemotherapy control group, our results should be interpreted cautiously. Nevertheless, the inclusion of patients with various genetic drivers, such as KRAS, ALK, MET, and HER2, allows our study to contribute exploratory data regarding the potential benefit of ABCP in these subgroups. Furthermore, recent real‐world studies have shown that ABCP can achieve favorable disease control in patients with various genetic drivers, although most of these reports primarily focused on *EGFR*‐mutated or wild‐type populations [29, 30].

Recent studies have provided additional insights into the efficacy of immunotherapy in NSCLC patients with genetic driver alterations other than EGFR mutations. Tian et al. conducted a multicenter real‐world analysis showing that tumors harboring *KRAS* mutations generally exhibited better responses to ICI‐based therapies compared to those with *ALK* or *ROS1* rearrangements [31]. Similarly, Garassino et al. reported that first‐line immunotherapy demonstrated only modest efficacy in patients with BRAF, MET, or HER2 alterations, with outcomes varying by mutation subtype [32].

While our study differs in treatment regimen and design, we observed that ABCP therapy showed modest activity across various driver‐positive subgroups, including KRAS, ALK, MET, and HER2. These observations warrant further investigation and should be interpreted cautiously due to the limited sample size and lack of a chemotherapy control group. Notably, PD‐L1 expression ≥ 50% was independently associated with longer PFS in our analysis. This suggests that PD‐L1 expression may have prognostic and predictive relevance even in patients harboring genetic alterations, highlighting the importance of considering PD‐L1 status when evaluating chemoimmunotherapy outcomes in this population.

This study has some limitations. First, it was a single‐center retrospective study with potential selection bias and confounding factors. Second, the relatively small sample size may reduce the statistical power to detect differences in specific genetic subgroups. Third, we did not include a driver‐negative control group, which restricts the generalizability of our findings. Therefore, our results should be interpreted as exploratory and hypothesis‐generating. Fourth, there was a significant imbalance in prior treatment exposure between groups, with most patients with EGFR mutation receiving ABCP after TKI failure, whereas many patients with other alterations received ABCP as first‐line therapy, although the IPASS trial suggested that the sequence of TKI and chemotherapy may not critically affect OS [33]. The effect of this imbalance on our results cannot be completely ruled out. Although treatment line was not significantly associated with PFS in univariable analysis and was therefore not included in the multivariable model, we acknowledge that this decision may still limit the interpretation of our findings.

These findings add to the body of evidence by including patients with KRAS, ALK, MET, HER2, other rare mutations, and the effect of ABCP therapy after EGFR resistance. Additionally, our findings highlight the potential role of ABCP in managing these heterogeneous genetic alteration cohorts. In conclusion, ABCP therapy showed activity in NSCLC with EGFR mutations and other genetic alterations, with no statistically significant PFS difference between groups. These results are exploratory and should be interpreted with caution.

## Author Contributions

**Tsunehiro Tanaka:** conceptualization, data curation, formal analysis, investigation, methodology, project administration, resources, validation, visualization, writing – original draft. **Motohiro Tamiya:** conceptualization, funding acquisition, investigation, methodology, project administration, resources, supervision, writing – original draft, writing – review and editing. **Akito Miyazaki:** investigation, resources, validation, writing – review and editing. **Kiyohide Komura:** investigation, resources, validation, writing – review and editing. **Shun Futamura:** investigation, resources, validation, writing – review and editing. **Takahisa Kawamura:** investigation, resources, validation, writing – review and editing. **Kei Kunimasa:** investigation, resources, validation, writing – review and editing. **Takako Inoue:** investigation, resources, validation, writing – review and editing. **Kazumi Nishino:** investigation, resources, validation, writing – review and editing.

## Conflicts of Interest

Dr. Tsunehiro Tanaka declares no conflicts of interest relevant to this study. Dr. Motohiro Tamiya has received consulting fees from Pfizer and honoraria for lectures or educational events from Pfizer, AstraZeneca, Eli Lilly, MSD, Chugai Pharmaceutical, ONO Pharmaceutical, Bristol Myers Squibb, Takeda Pharmaceutical, Boehringer Ingelheim, and Amgen. Dr. Akito Miyazaki has received honoraria for lectures or educational events from AstraZeneca and MSD. Dr. Kiyohide Komura declares no conflicts of interest relevant to this study. Dr. Shun Futamura declares no conflicts of interest relevant to this study. Dr. Takahisa Kawamura has received honoraria for lectures or educational events from Chugai Pharmaceutical and AstraZeneca. Dr. Kei Kunimasa has received honoraria for lectures and educational events from Chugai Pharmaceutical and AstraZeneca. Dr. Takako Inoue reports research grants or contracts provided to her institution from ONO Pharmaceutical, Taiho Pharmaceutical, MSD, AbbVie, Daiichi Sankyo, Amgen, Eisai, Sanofi, Janssen Pharmaceutical, Novartis, Pfizer, Eli Lilly Japan, Merck Biopharma, Takeda Pharmaceutical, Chugai Pharmaceutical, and Merus. She has also received royalties or license payments from AstraZeneca, Chugai Pharmaceutical, Bristol Myers Squibb, ONO Pharmaceutical, and MSD. Dr. Kazumi Nishino reports the following conflicts of interest: support for this manuscript from Chugai Pharmaceutical (provided to both the author and her institution); research grants or contracts to her institution from ONO Pharmaceutical, Taiho Pharmaceutical, Eli Lilly Japan, AbbVie, Daiichi Sankyo, Amgen, Eisai, Sanofi, Janssen Pharmaceutical, Novartis, Pfizer, Merck Biopharma, Takeda Pharmaceutical, AstraZeneca, Merus, Gilead Sciences, MSD, and Bayer; and honoraria for lectures or educational events from AstraZeneca, Nippon Boehringer Ingelheim, Eli Lilly Japan, MSD, Novartis, Pfizer, Merck Biopharma, Janssen Pharmaceutical, Bristol Myers Squibb, Nippon Kayaku, ONO Pharmaceutical, Takeda Pharmaceutical, Chugai Pharmaceutical, Amgen, and Daiichi Sankyo.

## Supporting information


**Data S1:** Supporting Information.

## Data Availability

The datasets generated and/or analyzed during the current study are not publicly available due to patient privacy restrictions.
