# Supplementary material for: Efficacy of Atezolizumab, Bevacizumab, Carboplatin, and Paclitaxel Therapy in Patients With Genetic Alterations in Non‐Small Cell Lung Cancer
Source: Thorac Cancer. 2025 Sep 17;16(18):e70162. doi: 10.1111/1759-7714.70162 (PMC12443488; doi:10.1111/1759-7714.70162)
Supplement: Supplementary file 1 — Data S1: Supporting Information. [file TCA-16-e70162-s001.docx]

Supplementary Figure 1. Patient flow diagram for study cohort selection


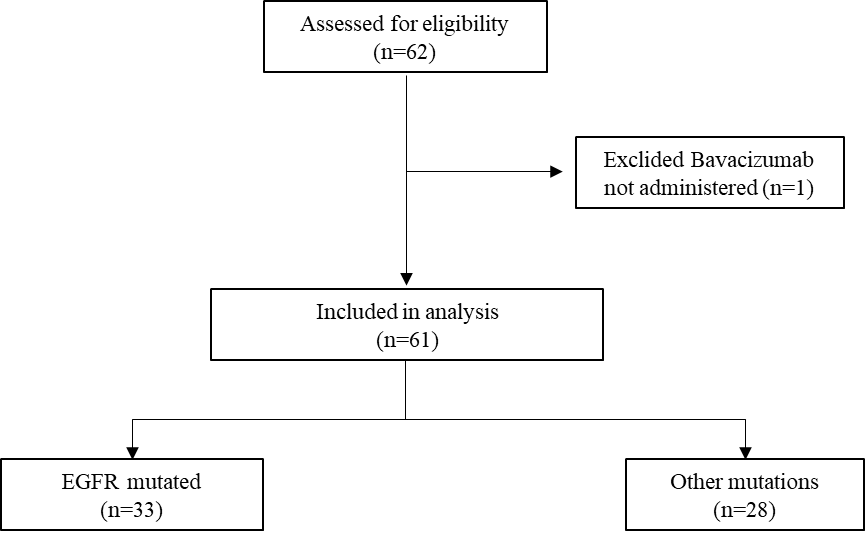


Of the 62 patients who received ABCP-based therapy during the study period, one patient was excluded due to omission of bevacizumab. The final analysis included 61 patients (EGFR mutated, n = 33; Other mutations, n = 28).

Supplementary Table S1. Genetic alteration subtypes in patients with non-small cell lung cancer (NSCLC)

| **Genetic alteration** | **Subtype** | **n (%)** |
| --- | --- | --- |
| EGFR (n=33) | Exon 19 deletion | 18 (54.5) |
|  | L858R | 11 (33.3) |
|  | L861Q | 1 (3.0) |
|  | L858R+E709X (compound) | 1 (3.0) |
|  | Exon 20 insertion | 1 (3.0) |
|  | L858R+T790M (compound) | 1 (3.0) |
| KRAS (n=14) | G12C | 9 (64.3) |
|  | G12V | 2 (14.3) |
|  | G12A | 2 (14.3) |
|  | G12P | 1 (7.1) |
| ALK (n=6) | Fusion | 6 (100) |
| MET (n=3) | Exon 14 skipping | 2 (66.7) |
|  | Amplification | 1 (33.3) |
| HER2 (n=1) | Exon 20 insertion | 1 (100) |
| BRAF (n=1) | V600E | 1 (100) |
| RET (n=1) | KIF5-RET fusion | 1 (100) |
| ROS1 (n=1) | Fusion | 1 (100) |
| NRG1 (n=1) | Fusion | 1 (100) |

Abbreviations: NSCLC, non-small cell lung cancer; EGFR, epidermal growth factor receptor; ALK, anaplastic lymphoma kinase; RET, rearranged during transfection.

These data are summarized in the Results section (Patients’ characteristics).

Supplementary Table S2. Response outcomes by genetic alteration status

| Response | EGFR mutated (n=33) | Other mutations (n=28) | P-value |
| --- | --- | --- | --- |
| CR | 0 (0.0) | 0 (0.0) | – |
| PR | 15 (45.5) | 14 (50.0) | – |
| SD | 9 (27.3) | 8 (28.6) | – |
| PD | 5 (15.2) | 5 (17.9) | – |
| NE | 4 (12.1) | 1 (3.6) | – |
| ORR (CR+PR) | 15 (45.5) | 14 (50.0) | 0.80 |
| DCR (CR+PR+SD) | 24 (72.7) | 22 (78.6) | 0.77 |

Percentages in the main text are shown for the primary analysis excluding non-evaluable (NE) cases; this table additionally provides a sensitivity analysis including NE as non-responders.

Abbreviations: ORR, objective response rate; DCR, disease control rate; CR, complete response; PR, partial response; SD, stable disease; PD, progressive disease; NE, not evaluable.

Supplementary Table S3. Detailed adverse events by genetic alteration status

|  | **EGFR mutated (n=33)** | | **Other mutations (n=28)** | |
| --- | --- | --- | --- | --- |
| **Adverse events** | **Any grade** | **Grade ≥3** | **Any grade** | **Grade ≥3** |
| **Hematologic toxicities** |  |  |  |  |
| Neutropenia | 22 (66.7) | 10 (30.3) | 14 (50.0) | 7 (25.0) |
| Anemia | 13 (39.4) | 1 (3.0) | 14 (50.0) | 2 (7.1) |
| Thrombocytopenia | 22 (66.7) | 5 (15.2) | 21 (75.0) | 1 (3.6) |
| Febrile neutropenia | 4 (12.1) | 4 (12.1) | 2 (7.1) | 2 (7.1) |
| **Non-hematologic toxicities** |  |  |  |  |
| Fatigue | 5 (15.2) | 2 (6.1) | 4 (14.3) | 0 (0.0) |
| Alopecia | 11 (33.3) | 2 (6.1) | 11 (39.3) | 2 (7.1) |
| Peripheral neuropathy | 10 (30.3) | 4 (12.1) | 14 (50.0) | 2 (7.1) |
| Proteinuria | 15 (45.5) | 3 (9.1) | 11 (39.3) | 4 (14.3) |
| Hypertension | 2 (6.1) | 0 (0.0) | 3 (10.7) | 2 (7.1) |
| Anorexia | 1 (3.0) | 1 (3.0) | 4 (14.3) | 0 (0.0) |
| Constipation | 6 (18.2) | 0 (0.0) | 3 (10.7) | 1 (3.6) |
| Diarrhea | 1 (3.0) | 1 (3.0) | 1 (3.6) | 1 (3.6) |
| Mucositis | 1 (3.0) | 0 (0.0) | 1 (3.6) | 0 (0.0) |
| Fever | 5 (15.2) | 3 (9.1) | 1 (3.6) | 1 (3.6) |
| AST elevation | 19 (57.6) | 8 (24.2) | 15 (53.6) | 5 (17.9) |
| ALT elevation | 17 (51.5) | 7 (21.2) | 14 (50.0) | 5 (17.9) |
| **Immune-related AEs (irAEs)** |  |  |  |  |
| Any irAEs | 13 (39.4) | 5 (15.2) | 13 (46.4) | 5 (17.9) |
| Rash | 5 (15.2) | 2 (6.1) | 9 (32.1) | 2 (7.1) |
| Pneumonitis | 0 (0.0) | 0 (0.0) | 2 (7.1) | 2 (7.1) |
| Meningitis | 3 (9.1) | 3 (9.1) | 0 (0.0) | 0 (0.0) |
| Guillain–Barré syndrome | 1 (3.0) | 1 (3.0) | 0 (0.0) | 0 (0.0) |
| Hypothyroidism | 2 (6.1) | 0 (0.0) | 3 (10.7) | 1 (3.6) |
| Type 1 diabetes mellitus | 1 (3.0) | 1 (3.0) | 0 (0.0) | 0 (0.0) |
| Adrenal insufficiency | 1 (3.0) | 0 (0.0) | 0 (0.0) | 0 (0.0) |
| Colitis | 1 (3.0) | 0 (0.0) | 0 (0.0) | 0 (0.0) |

Summary frequencies of adverse events are provided in Table 3 of the main text; this table presents a detailed breakdown by event type and grade.

Abbreviations: AE, adverse event; irAEs, immune-related adverse events; EGFR, epidermal growth factor receptor.
